# Retention in Telehealth Treatment for Opioid Use Disorder Among Rural Populations: A Retrospective Cohort Study

**DOI:** 10.1089/tmj.2023.0044

**Published:** 2023-12-08

**Authors:** Marlene C. Lira, Cynthia Jimes, M. Justin Coffey

**Affiliations:** ^1^ Workit Labs, Workit Health, Ann Arbor, Michigan, USA.; ^2^ Geisinger Commonwealth School of Medicine, Scranton, Pennsylvania, USA.

**Keywords:** *telemedicine*, *telehealth*, *substance use disorder*, *opioid use disorder*, *rural*, *buprenorphine*

## Abstract

**Introduction:**

*There are limited studies to date on telemedicine treatment outcomes for opioid use disorder (OUD) among rural populations.*

**Methods:**

*This was a retrospective cohort study of rural adults enrolled in telemedicine OUD treatment. Study outcomes were percent retained in care and adherence to buprenorphine assessed by urine drug screens at 1, 3, and 6 months.*

**Results:**

*From April 1, 2020, through January 31, 2022, 1,816 rural patients across 14 states attended an initial telemedicine visit and received a clinical diagnosis of OUD. Participants had the following characteristics: mean age 37.7 years (±8.6); 52.4% female; and 66.7% Medicaid. At 1, 3, and 6 months, 74.8%, 61.5%, and 52.3% of participants were retained in care, and 69.0%, 56.0%, and 49.2% of participants were adherent, respectively.*

**Conclusions:**

*Telemedicine is an effective approach for treating OUD in rural populations, with retention comparable to in-person treatment.*

## Introduction

The opioid epidemic in the United States disproportionately affects rural communities and researchers have called for evaluations of innovative models of care that address these disparities.^1–6^ Given that telemedicine may increase treatment access, privacy, and flexibility, while overcoming barriers such as long commutes, it is a particularly promising model of care for delivering opioid use disorder (OUD) treatment to rural populations.^7,8^ Research has indicated that rural patients are more likely to utilize telemedicine for OUD treatment than their nonrural counterparts, and that COVID-era expansions in telehealth increased enrollment for OUD treatment by telemedicine among individuals in rural areas.^9–11^

Although telemedicine models of care for individuals with OUD are an effective alternative to traditional in-person care,^12–14^ few studies have examined outcomes of telemedicine-delivered OUD treatment among patients living in rural areas specifically.^9,15,16^ Therefore, we performed an analysis to assess retention in care and medication adherence among individuals residing in rural areas and receiving telemedicine treatment of OUD.

## Methods

### STUDY DESIGN, SETTING, AND PARTICIPANTS

This was a retrospective cohort study of individuals residing in rural areas, who initiated OUD treatment at Workit Health, a telemedicine provider of treatment for substance use disorders. Workit Health's clinical program follows a “virtual first” model of care that includes video visits with providers and counselors, e-prescribing for substance use disorders and comorbid conditions, individual and group recovery counseling, and supportive care navigation and management. Patient encounters take place through a HIPAA-compliant video platform in the Workit Health mobile application or website.

All participants were at least 18 years old, resided in a U.S. Department of Agriculture (USDA)-designated rural zip code (Rural Urban Commuting Area code ≥4),^17^ attended at least one medical appointment through Workit's mobile application or web-based platform between April 1, 2020, and January 31, 2022, received a clinical diagnosis of OUD, and were offered buprenorphine and recovery counseling support. There were no exclusion criteria. A secondary analytic subgroup comprised individuals who provided self-reported characteristics at intake. We engaged with an independent, external IRB (Solutions IRB; FWA00021831), and the protocol was approved under expedited review with a waiver of informed consent, given this was a retrospective cohort study involving data collected solely for nonresearch purposes, in line with federal regulations (protocol no. 2022/08/16, approved October 26, 2022).

### TELEMEDICINE MODEL OF CARE FOR OUD

Patients can self-refer and enroll through Workit Health's phone-based application or website; patients learn of the program through online advertising, word-of-mouth, or a referring health care provider or organization. During an initial video visit for OUD, a full medical and social history is taken, diagnostic criteria for OUD and current opioid withdrawal are assessed, and treatment options are discussed. Buprenorphine-based medications were offered to all patients. As part of a patient-first approach, which is rooted in a harm reduction philosophy, neither detoxification nor abstinence is required for patients to receive treatment. If patients are not actively in withdrawal at the time of the visit, they are given instructions to initiate treatment with buprenorphine at a later time. Intranasal naloxone is prescribed to patients receiving buprenorphine as a preventive measure. Medical visits typically occur weekly in the first month, biweekly in the second month, and once per month thereafter.

During provider sessions, the provider reviews each patient's recent drug use and urine toxicology results and supports efforts to reduce substance use and adhere to medication as prescribed. Providers treat comorbid physical and mental conditions as indicated, desired, and feasible through telehealth (e.g., hepatitis C). Patients are asked to complete bloodwork at an outside laboratory in the first weeks of treatment to assess general health, as well as infectious disease status (e.g., HIV, hepatitis C). Given that internet connectivity issues may pose problems, patients receive instructions to test their internet connection before the first visit and clinicians are alerted in advance if poor connectivity could be an issue.

If patients do not have a stable internet connection during a visit, they are connected to a technology support specialist to troubleshoot. Patients are also able to switch to an audio-only/low-bandwidth video visit (i.e., by turning their video off) at any time during an appointment with one click. If the connection cannot be improved, the patient is rescheduled and the technology support specialist tries to find the source of the problem (e.g., the patient's device is not supported or there is insufficient bandwidth). Urine drug screens (UDS) are offered through a live, proctored option, as well as an automated option. UDS are conducted in strict compliance with state and federal regulations, and the timing therefore varies based on state requirements, but are generally conducted monthly after patients are stabilized in care.

As part of Workit Health's telehealth model of care, patients also have access to video-based counseling sessions, virtual counselor-led and mutual support groups, web-based cognitive behavioral therapy (CBT) courses, and convenient access to chat messaging with providers. Patients gain access to a digital CBT curriculum, which consists of ∼24 modules, which are delivered and deployed through Workit Health's web and phone applications. Patients are asked to complete at least two modules per week. Providers also recommend digital CBT modules based on relevant topics and immediate patient needs. In addition, participants engage with individual and group counseling sessions and online support groups as desired.

### OUTCOMES AND ANALYSIS

We defined retention in care at 1, 3, and 6 months as attending at least one visit within 15–45, 76–106, and 167–197 days of the initial visit, respectively, and was assessed in all patients with an initial visit, whether they subsequently withdrew from care or were lost to follow-up. Treatment adherence was defined as UDS positivity for buprenorphine at each treatment interval. Descriptive characteristics were calculated across the study sample for sex, age, insurance status, and rural designation. The percent of patients retained in care and the percent of patient adherent to buprenorphine were calculated at each time point, for which incomplete UDS were considered negative for buprenorphine. In addition, adherence was calculated among those retained in care and who completed UDS at each time point.

Bivariate differences in 6-month retention for sex, age, rurality, and insurance status were assessed in the main sample by *t*-tests, chi-squared tests, or Fisher's exact tests, as appropriate. Differences in 6-month retention were assessed for additional factors (education level, employment status, housing status, relationship status, and sexual orientation) among a subset of individuals who provided more extensive self-reported information.

## Results

**Fig. 1.**
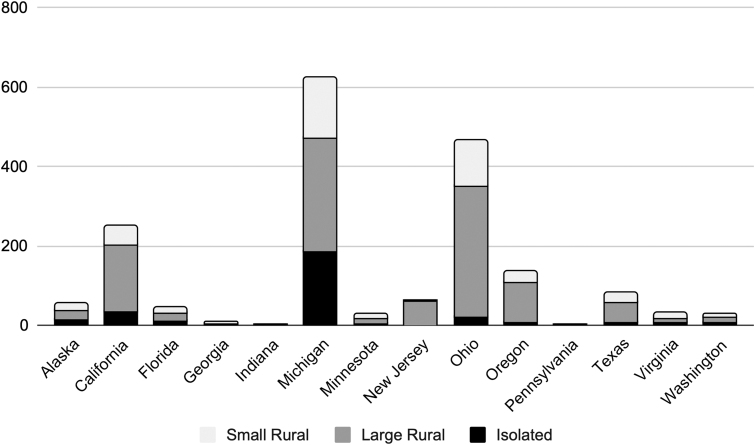
Geographic distribution of patients living in rural areas and receiving telemedicine treatment for opioid use disorder, Workit Health, 2020–2022 (*n* = 1,816).

From April 1, 2020, to January 31, 2022, 1,816 patients residing in rural zip codes attended an initial visit for treatment of OUD and met other eligibility criteria to comprise the study sample. Participants resided in 14 states during the study period, with the largest samples of patients located in Michigan (*n* = 624), Ohio (*n* = 464), and California (*n* = 252) (*Fig. 1*). USDA designations for the study population were as follows: 59.9% large rural, 23.2% small rural, and 16.9% isolated. The study population was 52.4% female with a mean age of 37.7 years (standard deviation 8.6), and 66.7% received Medicaid (*Table 1*).

**Table 1.** Characteristics of Patients in Rural Areas Receiving Telehealth Opioid Use Disorder Care and Retention in Care at 6 Months, Workit Health, 2020–2022

MAIN SAMPLE		6-MONTH FOLLOW-UP		
	OVERALL	RETAINED IN CARE	NOT RETAINED IN CARE	***p***
	***n*** = 1,816	***n*** = 949	***n*** = 867	
Sex				
Female	931 (52.4%)	488 (52.2%)	443 (52.6%)	0.63
Male	844 (47.5%)	445 (47.6%)	399 (47.4%)	
Other	1 (0.1%)	0 (0.1%)	0 (0.0%)	
Mean age (SD)	37.7 (8.6)	38.6 (8.7)	36.7 (8.4)	**<0.001**
Rurality				
Large rural	1,087 (59.9%)	539 (56.8%)	548 (63.2%)	**0.018**
Small rural	422 (23.2%)	234 (24.7%)	188 (21.7%)	
Isolated	307 (16.9%)	176 (18.5%)	131 (15.1%)	
Insurance status				
Commercial	251 (13.8%)	179 (18.9%)	72 (8.3%)	**<0.001**
Grant	2 (0.1%)	1 (0.1%)	1 (0.1%)	
Medicaid	1,212 (66.7%)	601 (63.3%)	611 (70.5%)	
Medicare	85 (4.7%)	56 (5.9%)	29 (3.3%)	
Multi-coverage	23 (1.3%)	17 (1.8%)	6 (0.7%)	
Self-pay	243 (13.4%)	95 (10.0%)	148 (17.1%)	

**SUBSAMPLE WITH ADDITIONAL CHARACTERISTICS**	**OVERALL**	**RETAINED IN CARE**	**NOT RETAINED IN CARE**	***p***
	***n* = 462**	***n* = 246**	***n* = 216**	
Education				
Less than high school diploma	85 (18.4%)	41 (16.7%)	44 (20.4%)	0.22
High school diploma	334 (72.3%)	176 (71.5%)	158 (73.1%)	
Associate's degree/vocational degree	33 (7.1%)	22 (8.9%)	11 (5.1%)	
Bachelor's degree or above	10 (2.2%)	7 (2.8%)	3 (1.4%)	
Employment				
Part time Full-time work/student	209 (45.2%)	131 (53.3%)	78 (36.1%)	**<0.001**
Retired/other	24 (5.2%)	10 (4.1%)	14 (6.5%)	
Unemployed/disabled	229 (49.6%)	105 (42.7%)	124 (57.4%)	
Housing				
Stable housing	356 (77.1%)	204 (82.9%)	152 (70.4%)	**<0.001**
Unstable housing	106 (22.9%)	42 (17.1%)	64 (29.6%)	
Partnered status				
Partnered	284 (61.5%)	167 (67.9%)	117 (54.2%)	**0.003**
Single	178 (38.5%)	79 (32.1%)	99 (45.8%)	
Sexual orientation				
Heterosexual/straight	426 (92.2%)	232 (94.3%)	194 (89.8%)	0.072
LGBTQIA+	36 (7.8%)	14 (5.7%)	22 (10.2%)	

Bold values indicate statistical significance at the 0.05 level.

**Fig. 2.**
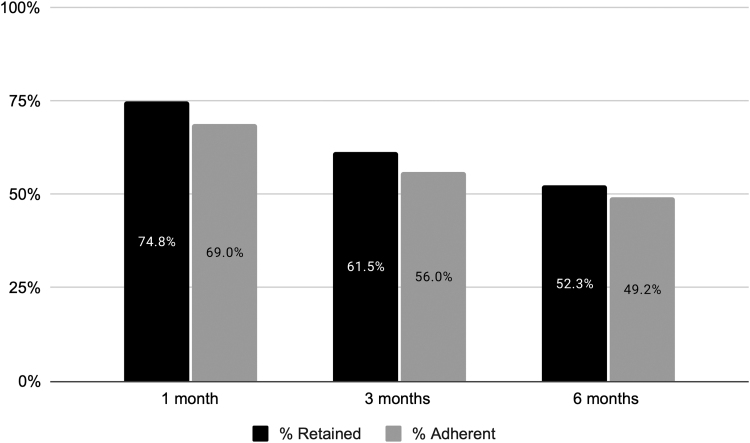
Retention and adherence by time point among patients living in rural areas and receiving telemedicine treatment for opioid use disorder, Workit Health, 2020–2022 (*n* = 1,816).

Retention in care at 1, 3, and 6 months was 74.8%, 61.5%, and 52.3%, respectively (*Fig. 2*). Adherence to buprenorphine at 1, 3, and 6 months was 69.0%, 56.0%, and 49.2%, respectively. Among those who were retained in care and completed a UDS at each time point, medication adherence at 1, 3, and 6 months was 98.6%, 98.9%, and 98.8%, respectively (not shown).

Within the main sample, there were significant differences in retention status at 6 months by age, rurality, and insurance status (*Table 1*), with individuals who were older, more rural, and covered by commercial insurance or Medicare more likely to be retained. Among a subgroup of patients with additional self-reported characteristics (*n* = 462), there were significant differences in retention status at 6 months by employment, housing, and relationship status.

## Discussion

This study examines retention in care and adherence to buprenorphine among rural patients receiving treatment for OUD through telemedicine across 14 states from April 2020 through January 2022, and explores factors associated with retention in care. This is the first study since the start of the COVID-19 pandemic to assess telemedicine OUD treatment outcomes among rural Americans specifically, and, to our knowledge, presents telemedicine OUD outcomes from the largest sample of rural Americans to date. We found retention in telemedicine-delivered care for OUD to be comparable to in-person treatment and found high rates of adherence to buprenorphine, as assessed through UDS.

In this rural, majority Medicaid sample, 75% of patients were retained in care and 69% were positive for buprenorphine at 1 month; a study using a nationally representative claims database of commercially insured patients found 1-month retention to buprenorphine/naloxone to be 69% and 1-month retention to buprenorphine to be 42%.^18^ We found that over 60% of patients were retained in care and 56% were positive for buprenorphine at 3 months, and about half were retained or positive for buprenorphine at 6 months. Although in-person retention outcomes at 3 and 6 months vary substantially,^19–24^ studies using all-payer pharmacy claims data and Medicaid claims data have found 6-month retention rates to be 35–41%.^24–26^

Adherence to buprenorphine at each time point, indicating an additional metric by which to assess compliance with recommended treatment, was high, with ∼90% of retained patients having UDS results positive for buprenorphine at each interval. Furthermore, among those patients retained in care and who completed UDS, adherence to buprenorphine was nearly universal.

Furthermore, these findings suggest higher retention than most previous studies of telemedicine OUD treatment, which have been limited to small programs (*n* < 500), mixed rural and nonrural populations, or non-U.S. populations.^1,15,27–32^ In our sample, over 60% of patients were retained at 3 months, which is higher than previously reported rates of 50%, with Weintraub et al. examining OUD treatment retention through telemedicine in a rural population specifically, and Zheng et al. comparing retention in OUD care between in-person and telemedicine modalities.^15,27^

In a study involving 58 clinics and both rural and urban patients in Ontario, Canada, retention in telemedicine OUD treatment (which also included methadone) at 6 months was 63%, higher than our observed rate of 52%.^28^ A report by Williams et al. recently explored 3-month retention in telemedicine OUD care, but patients were excluded from analyses if they were lost to follow-up within the first week, making direct comparisons challenging.^33^

Although the demographic characteristics we were able to report were limited in scope, they add to existing evidence on patients who engage in telemedicine for OUD care. Our cohort mostly was comprised individuals in their 30s and 40s, two-thirds of whom were covered by Medicaid. Frost et al. recently found that veterans who were younger, male, minority race, and with certain comorbidities were less likely to receive telehealth; notably, they found no difference in telehealth utilization by rural status.^12^

In bivariate analyses assessing differences in groups retained and not retained in care, we found that individuals who were retained in care were more likely to be older, living in isolated or small rural areas, partnered, have stable housing, or have commercial insurance or Medicare. Partnered status and housing stability are known protective factors for retention in OUD treatment.^23,34^ Although increasing age has previously been associated with lower odds of telemedicine receipt, it has also been found to be a protective factor for retention in OUD treatment.^12,23,34^

Our findings are important for several reasons. First, they add to the mounting evidence that treatment for OUD delivered entirely through telemedicine is feasible and effective.^12,13^ Second, our findings highlight the potential for telemedicine to expand access for rural communities and reverse disparities caused by treatment deserts. Third, given Workit Health's orientation to harm reduction principles and focus on low barrier care, our findings reinforce previous evidence that low-threshold treatment approaches may lead to higher retention rates.^35^ Finally, they illustrate the importance of permanent policy change for the continued use of telemedicine for treatment of OUD, without which many rural individuals struggling with OUD may be directly harmed.

These findings apply to a single organization's patient population and were limited by incomplete demographic data, which precluded the use of more robust regression models to assess factors associated with retention in care. Since the conclusion of the study period, additional assessments have been implemented and multivariable regression analyses should be implemented in the future. This research was conducted across multiple states and policy environments, some of which had small samples. Future research is needed to understand how state differences might relate to retention rates for addiction treatment delivered through telehealth.

In conclusion, in this retrospective cohort analysis of individuals living in rural areas and receiving telehealth treatment of OUD, over half of patients were retained at 6 months and adherence to buprenorphine was very high. Although our cohort was younger overall, older age was associated with retention in care, as were increasing rurality, housing stability, commercial/Medicare insurance, and partnered status. This report provides previously unavailable data specific to the success of telehealth care at reaching rural Americans with OUD—individuals whose ability to remain engaged in care would be compromised by restrictions on safe and effective telehealth practices.
